# Reversible dilative cardiomyopathy after electrical injury: a case report

**DOI:** 10.1186/s13256-018-1861-2

**Published:** 2018-11-08

**Authors:** Eirini Liodaki, Virginia Galati, Martin Bethge, Wolfgang Göpel, Peter Mailaender, Felix Stang

**Affiliations:** 1 Department of Plastic, Hand Surgery and Burn Care Unit, University Hospital Schleswig-Holstein, Campus Lübeck, Ratzeburger Alle 160, Lübeck, Germany; 2 Pediatric Cardiology, Paracelcus Health Center, Lübeck, Germany; 3 Pediatric Department, University Hospital Schleswig-Holstein, Campus Lübeck, Lübeck, Germany

**Keywords:** Reversible dilative cardiomyopathy, Dilative cardiomyopathy, Cardiomyopathy and electrical injury, Cardiomyopathy and burns

## Abstract

**Background:**

Dilative cardiomyopathy is an uncommon cardiac complication of electric shock.

**Case presentation:**

We report a case of a 12-year-old German boy with a high voltage injury who developed a four-chamber dilative cardiomyopathy, which was diagnosed on the 13th week postburn. One year after the accident, echocardiography showed a normal function of his heart with 64% ejection fraction and normal cavities’ dimensions.

**Conclusions:**

Despite the fact that dilative cardiomyopathy is not very common in electrical injuries but can be fatal, a prolonged echocardiography follow-up for patients with electrical injury could be recommended. Until now this case is the first child with severe burns after electrocution, who developed a reversible dilative cardiomyopathy.

## Background

Electrical burns typically comprise only a small percentage (approximately 3–4%) of total admissions to burn care units [1, 2]. However, this type of injury is considered one of the most devastating injuries due to its high morbidity and mortality [3] and is also the most frequent cause of amputations in a burn care unit [2]. Electrotrauma is divided between higher and lower voltage injuries with a borderline of 1000 volts [4]. Low voltage injuries typically cause only local disabilities in the place of contact, while high voltage injuries usually induce local disabilities and extensive devastation of deep structures, along with systemic effects [4]. Up to 40% of serious electrical injuries are fatal. High-voltage electrical injuries are truly devastating and cause long-term morbidity in those who survive including extremity amputations, blindness, and renal failure.

The most common cause of death continues to be cardiac arrest after acute arrhythmias at the scene of the incident secondary to either asystolia or ventricular fibrillation [1]. Electricity has been documented to cause myocardial necrosis, infraction, dysrhythmia, and contractile dysfunction, all of which may be delayed as well as persistent [5].

Dilative cardiomyopathy (DCM) is an uncommon cardiac complication of an electric shock. Only three cases of DCM caused by electrical injury have been reported in the international literature.

We report a case of a young boy with a high voltage injury who developed a reversible four-chamber DCM.

## Case presentation

**Fig. 1:**
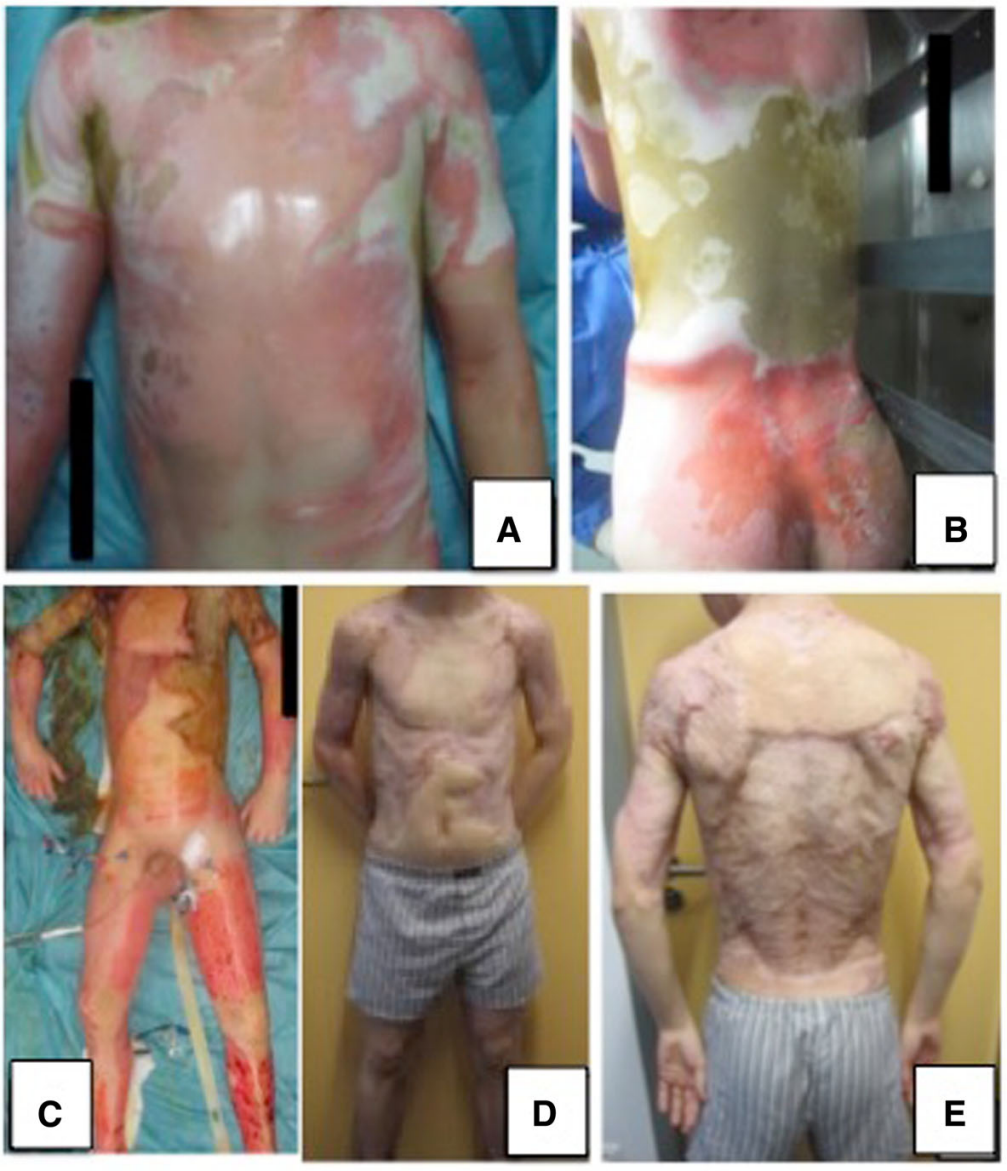
The 12-year-old boy was intubated at the site of the accident and immediately admitted to our burn care unit with deep partial-thickness and full-thickness burns. He sustained a 70% total body surface area burn of his face, neck, spine, thorax, abdomen, both arms, and both legs (**a**, **b**). Within the first 3 weeks, seven operations were performed; postoperative status 5 weeks (**c**) postburn. His appearance 18 months after the accident (**d**, **e**)

A 12-year-old German boy suffered from an accidental electrocution with 15,000 volts as he was playing in a railroad car. The boy was intubated at the site of the accident and immediately admitted to our burn care unit with deep partial-thickness and full-thickness burns. He sustained a 70% total body surface area (TBSA) burn of the face, neck, spine, thorax, abdomen, both arms, and both legs (Fig. 1a–c). A source lesion was noted on his right shoulder, and a ground lesion was visible on his right thigh. Directly after the admission, escharotomy and tracheostomy took place. In the first 24 to 48 hours after the removal of blisters a “wet-wound-dressing” with paraffin gauze dressing and polyhexanide solution was applied.

He was resuscitated according to the Parkland formula. In the first 24 hours, only Ringer lactate solutions and no colloids were used. He was started on a high-calorie diet (enteral feeding). Cardiac monitoring was done for 24 hours and no cardiac dysrhythmias were observed.

Within the first 3 weeks seven operations were performed including dermabrasion, application of Suprathel® (PolyMedics Innovations GmbH, Denkendorf, Germany), tangential excision and split-thickness skin graft, epifascial excision, application of Integra™ (Integra LifeSciences Corp., Plainsboro, NJ, USA), and autologous keratinocyte transplantation.

The duration of the mechanical ventilation reached 85 days.

In the course of the stationary treatment (135 days) he developed acute renal failure treated with veno-venous hemofiltration for 7 days and acute liver failure treated conservatively.

**Fig. 2:**
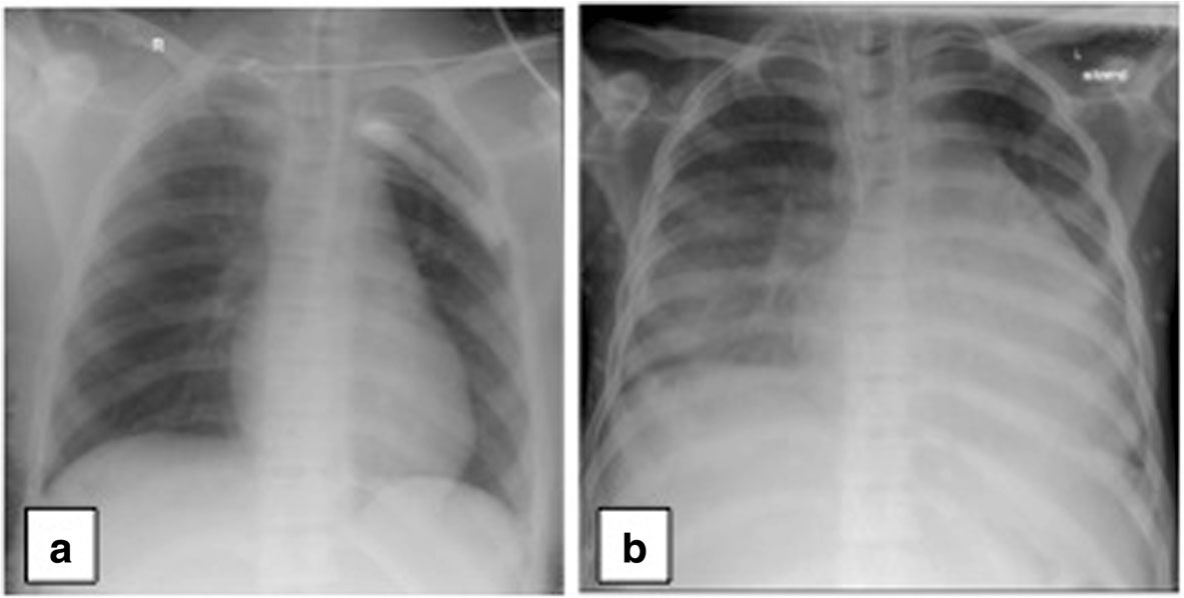
A chest X-ray showed an increase of the cardiothoracic ratio from 0.50 (**a**) at the time of admission to 0.63 (**b**) at the 10th week postburn

**Fig. 3:**
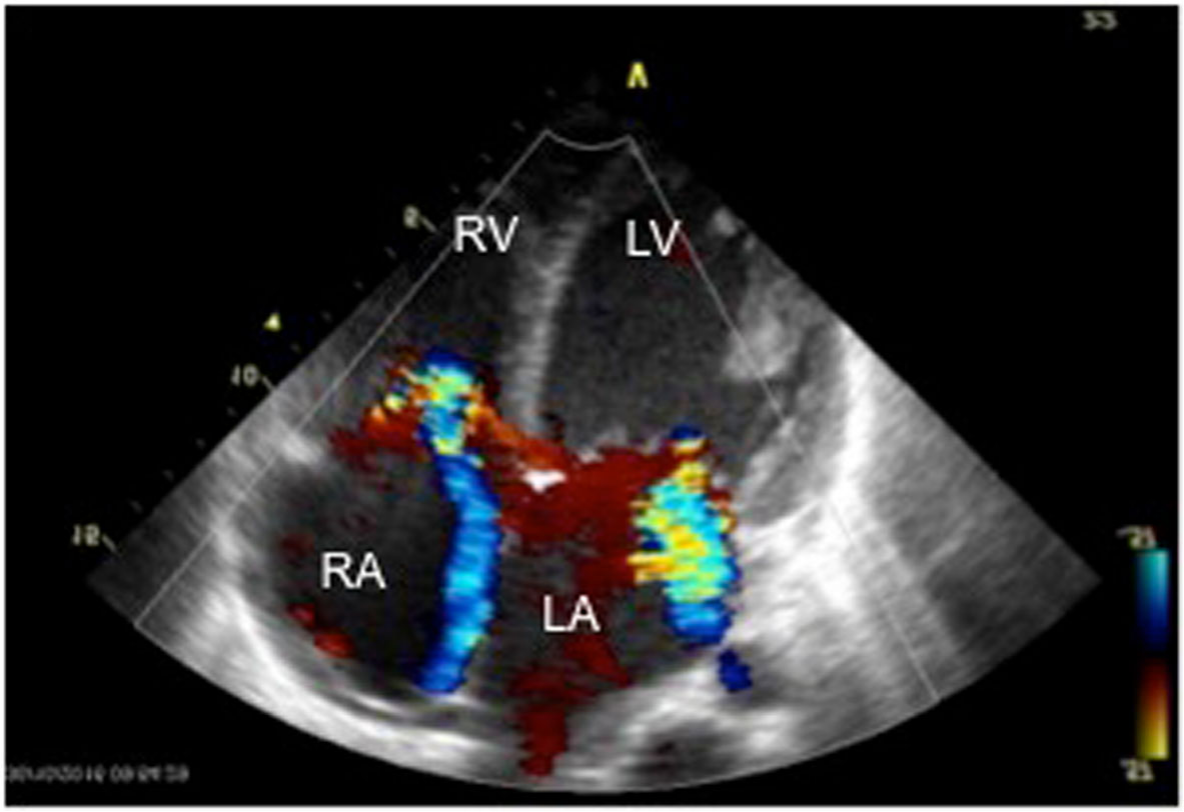
A four-chamber dilative cardiomyopathy with biventricular dysfunction (left ventricular ejection fraction 18%) was diagnosed 13 weeks after the accident. The echocardiography findings showed: enlargement of all the heart cavities; a left ventricular end diastolic diameter 65 mm (normal values 46.8 ± 6 mm); ejection fraction 18% (normal values 50–65%); tricuspid annular plane systolic excursion 1 cm (normal values > 1.6 cm); distinct central mitral insufficiency; and moderate tricuspid insufficiency. There was also a high suspicion of secondary pulmonary hypertension in the context of left ventricular insufficiency (systolic pulmonary artery pressure 45 mm Hg + central venous pressure). *LA* left atrium, *LV* left ventricle, *RA* right atrium, *RV* right ventricle

The boy developed persisting hypotension, edema, and ascites after the 10th week postburn. The hypotension required dobutamine therapy. A chest X-ray showed an increase of the cardiothoracic ratio from 0.50 (at the time of admission) to 0.63 (at this critical point) (Fig. 2). In order to clarify this persisting hypotension, a second echocardiography was performed. The first echocardiography was performed 4 weeks after the accident proving the healthy initial condition of the heart of our young patient. A four-chamber DCM with biventricular dysfunction was diagnosed 13 weeks after the accident: left ventricular ejection fraction (LV-EF) 18% (Fig. 3).

The most common possible causes of DCM (viruses, infections, drugs, toxins, endocrinologic disorders, metabolic disorders, and arrhythmia) were tested and excluded.

Inotropic therapy was initially required. It began with digitalis and then an application with phosphodiesterase inhibitor (milrinone) followed. A diuretic therapy with torasemide was also applied. Then, heart failure therapy followed with angiotensin-converting enzyme (ACE) inhibitor (enalapril), beta blocker, diuretics, and digoxin.

At the point of hospital discharge there was an increase of EF (22%) as well as an increase of the contractility of his heart. The LV end diastolic diameter (LVEDD) reached 58 mm.

One year after the accident, echocardiography showed a normal function of his heart with 64% EF and normal cavities’ dimensions (LVEDD 51 mm). No mitral and tricuspid insufficiency was present.

## Discussion

To the best of our knowledge, there are only three cases of DCM caused by electrical injury reported in the international literature. In these cases the victims were all adults aged 24–57 years without predisposing conditions. Two suffered from a high-voltage electrical injury (12,600 V and 13,000 V). One patient died on the third day postburn and an autopsy showed that the cause of death was severe heart failure by acute four-chamber dilated cardiomyopathy. The other two patients died within 18 months after the incident.

The pathophysiological mechanism of DCM secondary to electric shock is not clear. Myocardial dysfunction may be a complication of thermal/electrical injury [1]. This can initially be attributed to fluid shifts that occurred as a result of plasma loss into the burn area, producing a fall in venous return and a decrease in preload [1]. The electric shock could cause myocardial necrosis, myocardial infraction, arrhythmia, conduction disturbances as well as contractile dysfunction [1]. DCM appearing after an electrical injury could be a result of direct injury to the myocardium (contraction band necrosis) or a result of host’s inappropriate response to injury leading to a cytokine-induced myocardial dysfunction [1]. DCM could also be a result of over-resuscitation or cardiotoxic agents [6].

## Conclusions

Until now the above-mentioned case is the first child with severe burns after electrocution (15,000 volts), who developed a reversible DCM. Despite the fact that DCM is not very common in electrical injuries but can be fatal, a prolonged echocardiography follow-up for patients with electrical injury could be recommended.
